# Dietary Intake and Eating Behavior During Pregnancy by Pre-Pregnancy Nutritional Status

**DOI:** 10.3390/life16030479

**Published:** 2026-03-16

**Authors:** Małgorzata Szczuko, Justyna Kikut, Małgorzata Tomasik, Maciej Ziętek

**Affiliations:** 1 Department of Bromatology and Nutritional Diagnostics, Pomeranian Medical University, 71-460 Szczecin, Poland; 2 Supraregional Center for Parenteral and Enteral Nutrition for Children with the Clinic of Pediatrics, Nutrition and Pediatric Gastroenterology, University Clinical Hospital No. 1, Pomeranian Medical University, 71-460 Szczecin, Poland; justyna.kikut@pum.edu.pl; 3 Department of Interdisciplinary Dentistry, Faculty of Medicine and Dentistry, Pomeranian Medical University, 70-111 Szczecin, Poland; malgorzata.tomasik@pum.edu.pl; 4 Department of General Pharmacology and Pharmacoeconomics, Pomeranian Medical University, 71-460 Szczecin, Poland; maciej.zietek@pum.edu.pl

**Keywords:** pregnancy, obesity, nutrition, education

## Abstract

Background: Excess body weight before pregnancy is common and may be associated with suboptimal dietary intake and adverse metabolic outcomes. This study aimed to assess dietary intake and selected metabolic parameters in pregnant women with pre-pregnancy overweight or obesity compared with women of normal pre-pregnancy body weight, and to explore changes following nutritional education. Methods: The study included 62 pregnant women between 10 and 36 weeks of gestation. The study group consisted of 44 women with pre-pregnancy overweight or obesity, while the control group included 18 women with normal pre-pregnancy body weight. Dietary intake was assessed using repeated 24 h dietary recalls and analyzed before and after a nutritional education intervention. Nutrient intake was compared with national dietary reference values, and selected biochemical parameters were analyzed. Results: Before nutritional education, diets in both groups were characterized by a high proportion of energy derived from fat, excessive intake of saturated fatty acids (SFAs), sodium, and phosphorus, and insufficient intake of dietary fiber, vitamin D, folates, iron, and iodine. After nutritional education, modest changes in dietary composition were observed, including a reduction in SFA and sucrose intake and a slight shift toward a higher proportion of energy derived from protein. However, improvements in biochemical parameters were limited. Conclusions: In this exploratory study, short-term nutritional education during pregnancy was associated with modest dietary modifications but had a limited impact on metabolic parameters. The results do not support strong clinical recommendations or modifications of existing dietary guidelines but underscore the need for larger, well-powered studies with longer follow-up to better evaluate the effectiveness of nutritional interventions in pregnant women with different pre-pregnancy nutritional statuses.

## 1. Introduction

Analysis of data from many countries shows that obesity is a global epidemic. The prevalence of obesity exhibits a consistent upward trajectory, irrespective of ethnicity, race, age, or gender [1,2]. Excess body weight before pregnancy is highly prevalent worldwide and remains a major public health concern. According to the World Health Organization, approximately 40% of women of reproductive age are overweight and more than 15% are obese, with similarly high and increasing rates reported in Poland. Pre-pregnancy overweight and obesity are associated with adverse maternal and pregnancy outcomes, including gestational diabetes, hypertensive disorders, and impaired metabolic adaptation during pregnancy [3]. A higher BMI is closely associated with abdominal obesity, metabolic syndrome, insulin resistance and dyslipidemia. Dietary interventions during pregnancy have therefore received considerable attention. Recent well-designed randomized controlled trials have demonstrated that structured lifestyle interventions—including digital health tools, dietary pattern modification, and multidomain approaches—can improve selected maternal and offspring outcomes. For example, the HealthyMoms randomized controlled trial showed that a smartphone-based intervention supported healthier gestational weight gain and dietary behaviors during pregnancy [4]. The IMPACT BCN trial demonstrated that adherence to a Mediterranean diet or mindfulness-based stress reduction reduced the risk of small-for-gestational-age births in at-risk pregnancies [5]. More recently, the WINGS trial highlighted the long-term benefits of integrated nutritional and behavioral interventions initiated before conception and continued through early childhood on child neurodevelopmental outcomes [6].

Maternal obesity is associated with an increased risk of adverse pregnancy outcomes. The risk of early miscarriage is approximately 30% higher in women with obesity and may be doubled in those with the highest BMI categories [7]. The most common obesity-related pregnancy complications include gestational diabetes and preeclampsia [8], with gestational diabetes occurring four to nine times more frequently in women with obesity and affecting up to 25% of overweight women [7]. Elevated maternal BMI is also associated with a substantially increased risk of preeclampsia, hypertension, thromboembolic disease [9,10], and postpartum hemorrhage following cesarean section [11].

Maternal obesity is additionally linked to adverse offspring outcomes, including increased infant mortality, neonatal hypoglycemia [12], and a higher risk of congenital malformations, such as neural tube defects and cardiovascular anomalies [13,14]. Furthermore, obesity during pregnancy has been associated with impaired breastfeeding initiation and adverse long-term developmental and behavioral outcomes in children [12,15].

Evidence suggests that fetal programming is influenced by maternal nutritional status and dietary intake before and during pregnancy [16,17]. Current nutritional guidelines indicate that no additional energy intake is required in the first trimester, while energy needs increase in the second and third trimesters [18]. However, dietary recommendations vary internationally, emphasizing nutrient density rather than energy intake alone, particularly with respect to protein quality, essential fatty acids, and key micronutrients such as iron, iodine, calcium, and vitamin D [19,20,21]. In Canada, the emphasis is on adequate energy density and bioavailability of nutrients in the diet [16]. The German consensus emphasizes a small increase in energy requirements compared with a large increase in vitamin and mineral requirements. In Poland, nutritional standards are aligned with international recommendations but do not explicitly account for pre-pregnancy nutritional status or obesity [22,23].

Therefore, the aim of this study was to assess dietary intake and selected metabolic parameters in pregnant women with pre-pregnancy overweight or obesity compared with women of normal pre-pregnancy body weight, and to explore changes following short-term nutritional education. The study was designed as an exploratory analysis to examine whether nutritional education is associated with measurable changes in dietary composition and metabolic markers.

## 2. Material and Methods

### 2.1. Study Group and Dietary Intake

The study included 62 women who were between 10 and 36 weeks of gestation, who volunteered for the study. In the study group (SG), we distinguished 44 women, including 29 women with obesity (BMI > 30) and 15 overweight (BMI ≥ 25) before pregnancy. In the control group (CG), we included 18 women with normal weight (BMI 20-24.9) before pregnancy. It was a random sample of the inhabitants of the city and the surrounding area. Assuming a population of 1000 women, 95% confidence level, 0.8 fraction size and a maximum error of 10%, n = 58 women. The inclusion criterion was that the pregnancy had been confirmed by a biochemical test (Beta HCG) and high-resolution ultrasonography imaging (General Electric, Voluson E8 Expert, USA, 2015). The exclusion criteria were women with low BMI (<20) or advanced pregnancy (over 32 weeks only applied to the follow-up visit) and women under the age of 18 (the age of majority in Poland). Both groups received the same dietary recommendations (WHO) adjusted to their physiological condition and anthropometric parameters. After the 4–8-week period, the women were requested for a follow-up visit. A total of 49 women, including 31 with excessive body weight and 18 from the control group, attended the follow-up visit after receiving nutritional education. Some women in the SG did not attend the follow-up at a dietitian visit because their pregnancy had advanced beyond 32 weeks (n = 7), and several women had miscarriages (n = 4) or they did not show up for the tests (n = 2).

### 2.2. Methods for Collecting Dietary Intake Data

Dietary intake data were collected using the method of multiple dietary intake interviews, which covered the last 24 h. Four days of their diet were analyzed for each woman before joining the project, as well as the implementation of a new diet at the follow-up visit. The new diets met the requirements and implementation of nutritional standards using standard products found in the traditional diet of the Polish population and available imported products [18]. It was the standard procedure. All visits were carried out by two qualified dietitians with at least a doctorate degree. Dietary interviews were assessed using the Diet 6D diet program recommended by the Institute of Food and Nutrition. This program allows determination of dietary nutrient intake of proteins, including amino acids; fat (SCFA, MUFA, PUFA); carbohydrates (including sucrose, lactose, and starch); vitamins; minerals; dietary fiber; and cholesterol—a total of 58 macro- and micronutrients. The obtained data were compared to the binding Nutrition Standards for the Polish population [18].

To interpret the results, norms according to the mean age of the study group were used. They were based on the levels of the average group requirements (EAR) or, when they was not established, adequate intakes (AIs), which are presented in Table 1. The percentage of nutrient intake was calculated taking into account the Recommended Dietary Allowances (*RDAs*) or, when they were not available, AIs, which are presented in Table 2A,B. Reference levels for standards were calculated with an error margin of +/− 10%. Energy intake standards were calculated taking into account mean age, body weight, and height.

**Table 1:** The assumed nutritional norms.

**A. Evaluation of Nutrition with the Use of Estimated Average Requirement (EAR) Cut-Off Points**	
Protein (g)	44–78
P (mg)	580
Mg (mg)	360
Zn (mg)	9.5
Cu (mg)	0.8
Iodine (µg)	160
Vitamin A (µg)	530
Vitamin B1 (mg)	1.2
Vitamin B2 (mg)	1.2
Niacin (mg)	1.4
Vitamin B6 (mg)	1.6
Folates (µg)	520
Vitamin B12 (µg)	2.2
Vitamin C (mg)	70
**B. Evaluation of nutrition using the adequate intake (AI) level.**	
Na (mg)	1500
K (mg)	3500
Ca (mg)	800
Fe (mg)	23
Manganese (mg)	2
Vitamin D (µg)	15
Vitamin E (mg)	11
**C. Evaluation of nutrition with the use of the norm of nourishment.**	
Energy (kcal)	2000–2400 *
Protein (%)	10–20%
Fat (%)	20–35%
UFA (g)	14–16.8 **
Carbohydrates (%)	45–65%
Dietary fiber (g)	25

* energy calculated with reference to Nutrition Standards; ** UFA—unsaturated fatty acids.

**Table 2:** (**A**) Characteristics of the study and control groups at the beginning of gestation and during the first visit. (**B**) Characteristics of the study and control groups at the end of the 2nd trimester of pregnancy.

(**A**)			
**Feature Set**	**SG avg ± SD** n = 44	**CG avg ± SD** **n = 18**	***p***
**Anthropometric parameters**			
Age [years].	31.5 ± 5.6	32.2 ± 6.0	NS
Week of gestation [weeks]	18.8 ± 8.6	22.1 ± 8.3	NS
Height [cm].	1.7 ± 0.1	1.7 ± 0.1	NS
Pre-pregnancy weight [kg].	96.0 ± 16.0	62.6 ± 5.5	<10^−5^
Current weight [kg].	99.8 ± 15.2	65.6 ± 6.5	<10^−5^
BMI before pregnancy [kg/m^2^].	34.1 ± 6.2	21.7 ± 1.8	<10^−5^
BMI current [kg/m^2^].	35.5 ± 5.9	22.7 ± 1.9	<10^−5^
**Biochemical parameters**			
Total cholesterol [mg/dL].	197.8 ± 73.9	183.7 ± 53.6	NS
HDL cholesterol [mg/dL].	59.8 ± 23.7	67.5 ± 11.4	0.003
LDL cholesterol [mg/dL].	121.7 ± 52.7	124.2 ± 45.8	NS
Triglycerides [mg/dL].	155.2 ± 76.0	135.7 ± 55.1	NS
Fasting blood glucose [mg/dL].	86.5 ± 26.9	75.5 ± 14.8	0.002
Insulin [µIU/mL].	19.5 ± 18.1	13.5 ± 24.1	<10^−4^
HbA1c [%]	5.12 ± 1.6	4.80 ± 0.2	0.07 *
(**B**)			
**Feature set**	**SG avg ± SD** **n = 31**	**CG avg ± SD** **n = 18**	***p***
**Anthropometric parameters**			
Age [years].	32.6 ± 5.3	32.2 ± 6.0	NS
Week of gestation [weeks]	25.9 ± 5.6	26.5 ± 6.5	NS
Height [cm].	1.7 ± 0.1	1.7 ± 0.1	NS
Pre-pregnancy weight [kg].	92 ± 16.2	62.6 ± 5.5	<10^−4^
Current weight [kg].	97.3 ± 16.2	69.9 ± 7.2	<10^−4^
BMI before pregnancy [kg/m^2^].	31.9 ± 4.9	21.7 ± 1.8	<10^−6^
BMI current [kg/m^2^].	33.7 ± 4.7	24.2 ± 2.2	<10^−6^
**Biochemical parameters**			
Total cholesterol [mg/dL].	213.3 ± 30.0	187.9 ± 55.1	NS
HDL cholesterol [mg/dL].	56.2 ± 13.1	60.7 ± 11.2	NS
LDL cholesterol [mg/dL].	136.2 ± 26.8	152.4 ± 52.7	NS
Triglycerides [mg/dL].	211.2 ± 75.2	157.8 ± 42.0	0.045
Fasting blood glucose [mg/dL].	88.4 ± 5.7	78.2 ± 6.0	0.012
Insulin [µIU/mL].	18.8 ± 13.6	14.5 ± 15.6	NS
HbA1c [%]	5.02 ± 0.3	4.84 ± 0.3	0.036

(A) NS—not significant; *—trend; HbA1c— glycated hemoglobin. (B) NS—not significant; HbA1c—glycated hemoglobin.

### 2.3. Nutrition Education

Nutrition education for patients was conducted at the first visit at an average of 18.8 weeks of gestation in the SG and at 22.1 weeks in the CG. It focused on developing patients’ awareness of the need for adequate macronutrients and vitamins. Nutritional advice included the implementation of a low glycemic index and diet glycemic load and inclusion of healthy fat sources containing EPA/DHA. There was also a focus on sources of complete protein, nutrients promoting erythropoiesis, and an appropriate amount of green vegetables, especially those rich in folates. Attention was drawn to the necessity of limiting sources of simple sugars, including glucose-fructose syrup and highly processed food. The recommendations were based on the guidelines of the World Health Organization (WHO) and the Institute of Food and Nutrition [18]. In addition to dietary consultation, the patients were given a 14-day diet plan. The dietary consultation was conducted by two qualified dietitians (authors of this article), who examined their patients. Study participants found out about the appointment a few days before it happened, which made it possible to reduce the likelihood of the appointment interfering with their diet.

### 2.4. Statistical Analysis

Statistical analysis was performed using Statistica 13.3 software (Statsoft, Krakow, Poland). The aim of the analysis was to compare nutrient intake levels between the two groups. The mean consumption before and after dietary education was compared between the study groups (SG vs. CG). The normality of distribution of continuous variables was verified by the Shapiro–Wilk test. The Shapiro–Wilk test was selected due to its high statistical power and reliability in assessing normality, especially for small and medium-sized samples, which is consistent with the characteristics of the analyzed dataset. As most of the analyzed continuous variables were not normally distributed, the non-parametric Mann–Whitney U test was used. We utilized linear mixed models (IBM^®^ SPSS^®^ Statistics) followed by the maximum likelihood ratio test to deal with missing data and to search for interactions between groups (obesity disease/normal weight) and time (before/after education). The significance level was taken as *p* < 0.05. Primary dietary endpoints were defined a priori and included percentage of energy derived from fat, intake of saturated fatty acids (SFA), sucrose, dietary fiber, folate, vitamin D, iron, and iodine. All remaining dietary variables were considered secondary and exploratory.

## 3. Results

Baseline anthropometric and biochemical characteristics of the study group (SG) and control group (CG) are presented in Table 2A,B. As expected, women in the SG differed significantly from the CG with respect to body weight and BMI both before pregnancy and at follow-up. These differences persisted throughout the observation period.

At baseline, the SG exhibited significantly higher fasting glucose and insulin concentrations, whereas HDL cholesterol levels were higher in the CG (Table 2A). At follow-up, women in the SG continued to present higher fasting glucose and triglyceride concentrations compared with the CG (Table 2B). Detailed anthropometric and biochemical data are provided in Table 2A,B.

### Analysis of Diets Before and After Nutrition Education

The primary dietary outcomes focused on intake of saturated fatty acids (SFA), sucrose, dietary fiber, folate, vitamin D, iron, and iodine. The distribution of nutrient intake relative to dietary reference values is summarized in Table 3A–C.

Before nutritional education, the SG was characterized by frequent underconsumption of folate, vitamin D, iron, and iodine, as well as insufficient dietary fiber intake. Excessive intake of SFA was also common. After nutritional education, modest improvements were observed, including a reduction in SFA and sucrose intake and slight increases in selected micronutrients; however, intake of folate, vitamin D, iron, iodine, and dietary fiber generally remained below recommended levels.

**Table 3:** The nutritional percentages of women in the SG with reference to the norms [%].

**A. Nutritional Percentages of the SG with Reference to the Below RDA Norms.**					
Constituent	norm	BEFORE EDUCATION		AFTER EDUCATION	
		SG		SG	
		<RDA	>RDA	<RDA	>RDA
P (mg)	580	-	100	-	100
Mg (mg)	300	72.3	27.7	65	35
Zn (mg)	9.5	97.7	2.3	65	35
Cu (mg)	0.8	52.3	31.8	25	50
Ca (mg)	1000	100	-	90	10
Fe (mg)	23	97.7	2.3	100	-
Iodine (µg)	160	97.7	2.3	95	5
Vitamin A (µg)	530	18.2	65.9	30	55
Vitamin B1 (mg)	1.2	47.7	38.6	50	15
Vitamin B2 (mg)	1.2	65.9	15.9	25	65
Niacin (mg)	14	31.8	52.3	30	50
Vitamin B6 (mg)	1.6	45.5	45.5	35	20
Folate (µg)	520	97.7	2.3	100	-
Vitamin B12 (µg)	2.2	38.6	50	15	70
Vitamin C (mg)	70	43.2	47.7	45	40
**B. The nutritional percentages of the SG with reference to the below AI norms.**					
Constituent	norm	BEFORE EDUCATION		AFTER EDUCATION	
		SG		SG	
		<AI	>AI	<AI	>AI
Na (mg)	1500	4.5	81.8	10	90
K (mg)	3500	52.3	34.1	55	10
Manganese (mg)	2	9.1	84.1	-	85
Vitamin D (µg)	15	97.7	-	100	-
Vitamin E (mg)	10	47.7	38.6	60	20
**C. The nutritional percentages of the SG with reference to nutritional norms.**					
Constituent	norm	BEFORE EDUCATION		AFTER EDUCATION	
		SG		SG	
		<	>	<	>
Energy (kcal)	2400	59.1	18.2	100	-
Protein (%)	15–20	-	11.4	-	35
Fat (%)	20–35	2.3	31.8	-	5
SFA (g)	14–16.8	-	79.5	-	70
Cholesterol (mg)	300	-	27.3	45	35
Carbohydrates (%)	45–65	29.5	-	20	5
Dietary fiber (g)	25	52.3	27.3	55	15

Energy calculated with reference to nutrition standards.

Mean macronutrient intake before and after nutritional education is presented in Table 4A,B. Total energy intake did not differ significantly between groups at baseline. Prior to education, the SG exhibited higher sucrose intake and lower dietary fiber intake compared with the CG, while excessive SFA intake was observed in both groups.

Following nutritional education, a modest reduction in energy intake was observed, with a greater decrease in the SG. The proportion of energy derived from protein increased in both groups, while the proportion derived from fat decreased, particularly in the SG. SFA and sucrose intake declined in both groups; however, fiber intake remained insufficient in both the SG and CG.

After adjustment for multiple comparisons, several secondary dietary variables lost statistical significance, whereas all predefined primary endpoints retained their interpretation based on unadjusted analyses.

**Table 4:** The comparison of mean nutrient consumption (proteins including amino acids, fats, carbohydrates).

**A. BEFORE EDUCATION**			
Nutrient	SG avg ± SD n = 44	CG avg ± SD n = 18	*p*
Energy (kcal)	2168.6 ± 941.9	2180.8 ± 574.1	0.416
Total proteins (g)	89.1 ± 38.4	89.1 ± 25.6	0.614
Animal protein (g)	60.6 ± 32.6	58.9 ± 19.5	0.798
Plant protein (g)	28.2 ± 11.4	28.9 ± 10	0.540
Energy from protein (%)	16.8 ± 3.8	16.5 ± 3.7	0.932
Isoleucine (mg)	4301.3 ± 1919.4	4245.1 ± 1246.4	0.636
Leucine (mg)	6835 ± 3138.8	6689.5 ± 2059.4	0.625
Lysine (mg)	6096.9 ± 2782.8	6072.1 ± 1812.7	0.704
Methionine (mg)	2112 ± 949.2	2129.9 ± 640.8	0.500
Cystine (mg)	1250.4 ± 514.7	1298 ± 380.3	0.416
Phenylalanine (mg)	3885.9 ± 1689.5	3791.1 ± 1120.3	0.636
Tyrosine (mg)	3296.9 ± 1605.9	3073.3 ± 927.6	0.907
Threonine (mg)	3597.6 ± 1639.9	3565.3 ± 935	0.603
Tryptophan (mg)	1140.5 ± 513.2	1051.6 ± 295.1	0.786
Valine (mg)	5127.7 ± 2263.3	4943.5 ± 1380.4	0.798
Arginine (mg)	4077 ± 1855.8	4301.2 ± 1319.7	0.223
Histidine (mg)	2721.4 ± 1374.9	2747.7 ± 1212.8	0.762
Alanine (mg)	4176.7 ± 1764.9	4387 ± 1161.4	0.471
Aspartic acid (mg)	7575.6 ± 3165.1	7692.6 ± 2141.4	0.593
Glutamic acid (mg)	18,111.2 ± 7855.4	17,038.2 ± 6193.9	0.727
Glycine (mg)	3478.9 ± 1506.8	3611 ± 1103.1	0.561
Proline (mg)	6598.9 ± 3068.4	5894.3 ± 2250.3	0.443
Serine (mg)	4237.7 ± 1926.9	4115.7 ± 1160	0.625
Fat (g)	88.9 ± 46.1	92.4 ± 36.5	0.540
SFA (g) *	38.7 ± 22.6	31.7 ± 11.2	0.389
MUFA (g)	33.1 ± 20.3	36.2 ± 18.7	0.248
PUFA (g)	11.3 ± 8.2	15.3 ± 9.4	0.212
EPA (mg) *	0.04 ± 0.1	0.2 ± 0.5	0.212
DHA (mg) *	0.1 ± 0.2	0.6 ± 1.5	0.245
LCP (g)	0.1 ± 0.3	0.9 ± 2.4	0.306
Cholesterol (mg)	282.5 ± 173.1	326.1 ± 199.4	0.389
Energy from fat (%)	35.6 ± 9.9	36.3 ± 8.3	0.636
Total carbohydrates (g)	265.3 ± 128.5	275.6 ± 114.4	0.407
Assimilable carbohydrates (g)	242.1 ± 121.8	248.6 ± 106.6	0.603
Dietary fiber (g) *	21.1 ± 10.8	23.1 ± 11.9	0.160
Saccharose (g) *	53.5 ± 62.9	39.5 ± 25.4	0.727
Lactose (g)	12.2 ± 11.7	10.1 ± 10.3	0.636
Starch (g)	137.1 ± 62.3	127.7 ± 41.5	0.530
% energy from carbohydrates	45.5 ± 9.8	44.8 ± 8.3	0.582
**B. AFTER EDUCATION**			
Nutrient	SG avg ± SD (n = 31)	CG avg ± SD (n = 18)	*p*
Energy (kcal) *	1692 ± 319	2009.1 ± 496	**0.041**
Total proteins (g)	84.1 ± 18.2	90.2 ± 22.2	0.708
Animal protein (g)	58.8 ± 16.6	59.6 ± 16.7	0.194
Plant protein (g)	24.5 ± 5.5	29.1 ± 7.1	0.118
Energy from protein (%) *	19.7 ± 3.5	18.4 ± 3.6	**0.027**
Isoleucine (mg)	4062.5 ± 976.1	4288.6 ± 1112.5	0.416
Leucine (mg)	6459.4 ± 1421.4	6879.6 ± 1817.4	0.676
Lysine (mg)	5793.6 ± 1479.7	5961.8 ± 1642	0.495
Methionine (mg)	2040.8 ± 488.3	2151.4 ± 579.4	0.524
Cystine (mg)	1167.8 ± 271.4	1254.9 ± 282.5	0.344
Phenylalanine (mg)	3628.5 ± 824.6	3888.2 ± 970.7	0.391
Tyrosine (mg)	3034.9 ± 701.9	3218.5 ± 837.1	0.495
Threonine (mg)	3464.1 ± 883.1	3710.9 ± 986.3	0.676
Tryptophan (mg)	1080.6 ± 270.1	1131.6 ± 286.5	0.708
Valine (mg)	4821.2 ± 1087	5056.1 ± 1306.3	0.281
Arginine (mg)	4037.5 ± 1205	4592.9 ± 1420	0.644
Histidine (mg)	2415.9 ± 613.2	2511.2 ± 638.3	0.495
Alanine (mg)	4051.2 ± 1038.9	4323.6 ± 1205.9	0.644
Aspartic acid (mg)	7185.8 ± 1819.4	7698.7 ± 1997.6	0.226
Glutamic acid (mg)	16,025.9 ± 3322.7	17,685.3 ± 4245.8	0.468
Glycine (mg)	3351.2 ± 917.7	3655.2 ± 1096.2	0.210
Proline (mg)	5750 ± 1077.1	6199.7 ± 1495.9	0.391
Serine (mg)	3938.1 ± 937.3	4269.6 ± 1034.1	0.982
Fat (g) †	59.5 ± 20.2	74.9 ± 21.5	**0.033**
SFA(g) *	23.2 ± 8.1	28.5 ± 9.3	**0.038**
MUFA (g)	22.7 ± 9	27.8 ± 8.7	0.173
PUFA (g)	6.9 ± 3	9.5 ± 5.2	0.180
EPA	0.13 ± 0.1	0.27 ± 0.2	0.180
DHA	0.21 ± 0.3	0.38 ± 0.3	0.153
LCP (g)	0.2 ± 0.4	0.4 ± 0.4	0.262
Cholesterol (mg)	297 ± 126.8	338.9 ± 72.5	0.416
Energy from fat (%) *	30.7 ± 6.7	32.7 ± 5.3	0.553
Total carbohydrates (g)	216.1 ± 47.5	255.5 ± 76	0.082
Assimilable carbohydrates (g) †	194.3 ± 45.7	231.6 ± 71.6	**0.044**
Dietary fiber (g)	22.1 ± 7.4	24.9 ± 8.8	0.775
Saccharose (g) *	26.5 ± 12.5	28.8 ± 17.1	0.391
Lactose (g) †	9 ± 4.6	8 ± 4.7	**0.044**
Starch (g) †	109.4 ± 29.6	131.1 ± 29.8	**0.012**
Energy from carbohydrates [%]	46.6 ± 8.4	46.5 ± 7.2	0.582

SG—study group; CG—control group; bold—statistically significant differences; LCP—long-chain polyunsaturated fatty acids; * —*p* < 0.05, primary endpoint; † —*p* < 0.05, secondary endpoint, significant after FDR; primary endpoints are marked with an asterisk (*). For secondary endpoints, false discovery rate (FDR) correction using the Benjamini–Hochberg procedure was applied within the table; statistically significant results after FDR correction are marked with a dagger (†).

Vitamin and mineral intake before and after nutritional education is shown in Table 5A,B and Table 6A,B. At baseline, vitamin D and folate intake were critically low in both groups, while sodium and phosphorus intake exceeded recommended levels. Iron and iodine intake were insufficient, particularly in the SG.

After nutritional education, small changes in micronutrient intake were observed, including a slight increase in iodine intake in the SG and reductions in sodium and phosphorus intake in both groups. However, these changes were limited and generally did not result in the achievement of recommended intake levels. No clinically meaningful differences in mineral intake were observed between groups after education.

## 4. Discussion

There was no difference in dietary energy intake between the SG and CG before nutrition education. Interestingly, dietary energy intake was higher in the CG after nutrition education. Most et al. pointed out that women with excess body weight should not increase their energy intake during pregnancy [24]. In another study, a reduction in dietary energy intake was also observed in the control group after the intervention [25]. Nevertheless, both groups consumed insufficient dietary energy relative to their calculated requirements after the dietary intervention. This result may be due to the tendency of overweight women to underestimate their food portions [26,27,28]. However, these observations indicate that current dietary standards may require further evaluation with respect to their applicability to pregnant women with obesity.

### 4.1. Intake of Macronutrients, EPA, DHA and Fiber

A normal level of protein intake provided an adequate supply of amino acids. The ratio of animal to plant protein intake was around 2:1. In another study, lower protein and higher carbohydrate intakes were observed in the American population (North Carolina) compared to our results [29]. In contrast, McGowan et al. (Europe—Ireland) reported increased protein intake after dietary intervention, which is also supported by our observations [25].

The low intake of EPA and DHA at the beginning of pregnancy, also reported by other researchers [30], was improved by the dietary intervention. The dietary intervention reduced the intake of UFAs in both groups, especially in the SG. In addition, before the dietary intervention, the percentage of dietary fat was within the upper limits of the normal range, which was improved during nutritional education. An adequate intake of dietary fats, especially essential unsaturated fatty acids (EUFAs), is important because they cross the placenta and can affect the development of the amniotic membrane, among other effects. On the other hand, excessive dietary SFA intake may contribute to the development of lipid and carbohydrate metabolism disorders, including type 2 diabetes and gestational diabetes [31,32,33]. Similar beneficial dietary changes were observed in the study by Piirainen et al. [34] and Kinnunen et al. [35].

In our study, we found low fiber intake in both obese and normal-weight women, similar to the study by Dubois et al. [36]. Nutritional education slightly improved intake in our study. On the contrary, other researchers have observed a beneficial effect of intervention in increasing fiber intake [33,34]. With regard to pregnancy development, it is not only important to have an adequate supply of carbohydrates in the diet, but also its quality, i.e., the use of products with a low glycemic index (whole grains, non-starchy vegetables, berries, nuts, seeds, coarse-grained cereals) [37]. Fiber deficiency is still common, including in pregnant women, and the main reason is the elimination of the above food groups from the diet [38,39,40]. Adequate intake of dietary fiber can help reduce postprandial glycemia, with a beneficial effect on blood lipid levels in diabetic women, and prevent constipation. In addition, fiber delays the absorption of glucose, thereby increasing tissue sensitivity to insulin [41,42]. Fiber intake should therefore be emphasized in the education of this patient group. After applying the dietary intervention in our study, we were able to reduce dietary sucrose intake in both SG and CG, and in addition, the reduction in SG was statistically significant before and after for both groups. An excess of simple sugars in the diet, particularly sucrose, may contribute to increased birth weight of the baby [43].

### 4.2. Vitamin Intake

The vitamin A requirement was met in most of the women studied, contrary to the results of other researchers [44]. In terms of average intake, both groups consumed three times the reference value of vitamin A. Insufficient intake of vitamin E was observed in some of the women studied, which may contribute to pregnancy complications such as miscarriage, fetal growth restriction and preterm labor [45]. Insufficient intake of the above vitamins in American women has been reported by Hanson et al. [46]. Both before and after the nutritional education, drastically low vitamin D intakes were reported in almost 100% of subjects in both groups. Deficiency of this vitamin during pregnancy is associated with preeclampsia, allergies and multiple sclerosis in later life. Vitamin D deficiency can lead to reduced intestinal absorption of calcium [37]. Similarly, a study by Aparicio et al. showed that pregnant women are at risk of deficiencies in not only vitamin D but also in folic acid and iron, which is consistent with our study results. Other researchers have also confirmed the risk of a deficiency in these components in pregnancy [33].

In our study, we observed inadequate folate intake in both the SG and CG. All the women in the SG did not meet the RDA for this component. Other researchers have also observed inadequate folate intake in their studies [33,36,47]. Miyake et al. [48] also observed inadequate folate intake and recommended supplementation. Folic acid deficiency causes abnormal development of the fetal brain, spinal cord and neural tube, and folate deficiency during pregnancy is associated with emotional problems, schizophrenia and speech delays in offspring [49,50].

### 4.3. Mineral Intake

Calcium is one of the most important minerals for proper mineralization of fetal bones and for maintaining the health of the pregnant woman. It reduces the risk of pre-eclampsia and other hypertensive disorders [51,52]. Despite a normal intake of calcium in relation to average values, more than 50% of the women studied did not meet the standard for this component in terms of the percentage of their intake. Insufficient intake of calcium was observed in a study by Saunders et al. [33].

Iodine is an important nutrient during pregnancy, and its requirement increases by about 50% during pregnancy. Iodine deficiency can lead to congenital hypothyroidism, poor neuronal development, irreversible brain damage and intellectual disability, with a drop in IQ of almost 20 points and subsequent congenital hypothyroidism [53]. Iodine intake remained low in both study groups and improved slightly in the SG after nutritional education. In terms of percentage of intake, more than 90% of women in the SG were deficient in this element. Inadequate iodine intake was also observed in a study by Saunders et al. [33]. It therefore appears that dietary intake during pregnancy may be inadequate in many populations.

In our study, despite the introduction of the intervention, there was no increase in dietary iron intake. The inadequate intake of this element reached more than 90% in the SG. Similarly, other researchers have observed a deficit in this element in the diet of pregnant women [33,36,47]. Iron deficiency increases the incidence of perinatal mortality, preterm birth and low birth weight in newborns [54]. A study by Sunuwar et al. in anemic pregnant women showed that dietary intervention resulted in normalization of hemoglobin levels and an increase in iron-rich foods [55]. The body’s need for iron during pregnancy appears to be so high that supplementation is necessary.

Although a positive effect of education on sodium intake was reported, patients still consumed excessive amounts of sodium. Sodium intake was exceeded in more than 80% of women in both the SG and CG. High sodium intake has also been reported by other researchers [56,57]. However, pregnant women should be made aware of the complications that can result from an excessive intake of this element. Excessive sodium intake in the diet of pregnant women can lead to hypertension, which is considered to be the most common cause of maternal, fetal and neonatal death during labor [58].

In contrast, in the present study, we observed inadequate dietary potassium in both the SG and CG. Similarly, another study observed inadequate intake of this component in the diet [36]. The main dietary sources of potassium, such as bananas and potatoes, have a high glycemic index. Limiting their consumption is very useful during pregnancy, while including tomatoes, broccoli, chard, legumes, avocado, currants, nuts, fish and veal replenishes both potassium and other inadequately consumed nutrients such as iodine, selenium, iron, folates, PUFA, MUFA, EPA, DHA and vitamin D.

Diet has a major impact on the normal course of pregnancy and the health of the pregnant woman. In most studies, dietary errors are noted in the patients studied. It is therefore necessary to provide nutritional education to women who are planning to become pregnant and to those who are already pregnant [59,60].

### 4.4. Metabolic Parameters

With regard to biochemical tests, the group of pregnant European women studied by Harreiter et al. [61] had lower levels of fasting glucose and insulin, HDL and LDL cholesterol and triglycerides than the study group. It is known that due to hormonal changes during pregnancy (especially the increase in serum estrogen and progesterone levels), blood lipid levels increase with the duration of pregnancy [62,63,64,65].

The second and third trimesters of pregnancy are considered to be the diabetogenic period. Similar to the study group, increases in triglycerides, LDL cholesterol and carbohydrate metabolism parameters have been observed by other researchers [64,65]. At the same time, it should be noted that the dietary changes made allowed for relatively small changes in biochemical parameters in women with obesity compared to normal-weight women. The parameters that increased most were triglycerides, fasting plasma glucose and glycated hemoglobin. This confirms the previously suggested theory that improving the diet of women with obesity during pregnancy may not be enough to maintain metabolic parameters.

## 5. Limitations of the Study and Future Directions

The control group consisted of a limited number of women with normal pre-pregnancy body weight, which may have reduced the statistical power and limited the generalizability of the findings. Future studies should include a larger and more balanced sample across BMI categories, preferably in a multicenter design, to improve representativeness and robustness of comparisons. Moreover, there was limited assessment of lifestyle and confounding factors; physical activity, socioeconomic status, education level, and supplement use were not fully controlled. The intervention period (4–8 weeks) may have been insufficient to induce more pronounced and sustained changes in dietary habits or metabolic parameters. Longer-term interventions with repeated educational sessions and continuous dietary support may provide clearer insight into long-term dietary adherence and metabolic outcomes. Despite the implementation of nutritional education, the study design does not allow for direct cause-and-effect inference regarding the impact of dietary changes on metabolic parameters. Randomized controlled trials with standardized interventions are warranted to confirm causality and quantify the effectiveness of dietary education in different BMI groups. Furthermore, given the large number of comparisons performed across nutrients, the absence of correction for multiple testing substantially increases the risk of error.

## 6. Conclusions

This study provides an exploratory assessment of dietary intake and selected metabolic parameters in pregnant women with pre-pregnancy overweight or obesity compared with women of normal body weight, before and after nutritional education. The implemented dietary intervention was associated with modest changes in dietary composition, including a reduction in saturated fatty acid (SFA) and sucrose intake and a slight shift in macronutrient distribution. However, it was reported that biochemical parameters were limited.

These findings suggest that short-term nutritional education during pregnancy may lead to partial dietary modifications, but its impact on metabolic outcomes appears to be modest within the timeframe of this study. Importantly, the results should be interpreted with caution due to several limitations and a reliance on self-reported dietary intake methods, which are subject to recall bias and potential underreporting. Given the exploratory nature and limited magnitude of observed effects, the present results do not support strong clinical recommendations or modifications of existing dietary guidelines for pregnant women with overweight or obesity. Instead, they highlight the complexity of dietary behavior and metabolic regulation during pregnancy and underscore the need for larger, well-powered, and preferably randomized studies with longer follow-up periods. Future research should focus on comprehensive, longitudinal interventions that integrate dietary counseling with objective dietary assessment methods and account for potential confounding lifestyle and metabolic factors. Such studies are necessary to better understand the effectiveness of nutritional interventions during pregnancy and to inform evidence-based dietary recommendations for women with different pre-pregnancy nutritional statuses.

## Figures and Tables

**Table 5:** Vitamin consumption comparison.

**A. BEFORE EDUCATION**			
Vitamins	SG avg ± SD (n = 44)	CG avg ± SD (n = 18)	*p*
Vitamin A (µg)	1508.1 ± 2734.3	1132.8 ± 631.4	0.883
Retinol (µg)	486.2 ± 317.6	389.2 ± 229.7	0.364
Beta carotene (µg)	6206.9 ± 16,115.3	4495.1 ± 3704.6	0.636
Vitamin E (mg) †	11.4 ± 7.7	15.6 ± 9.4	0.073
Thiamine (mg)	1.2 ± 0.6	1.3 ± 0.4	0.071
Riboflavin (mg)	1.7 ± 0.8	1.7 ± 0.5	0.670
Niacin (mg)	19.4 ± 9.9	22.3 ± 10.9	0.317
Vitamin B6 (mg)	2 ± 0.8	2.4 ± 1	0.180
Vitamin C (mg)	88.7 ± 128	94.6 ± 98.4	0.647
Vitamin B12 (µg)	3 ± 1.5	4.5 ± 4.2	0.398
Vitamin D (µg) *	2.5 ± 3.1	5.3 ± 9.9	0.994
Folate (µg) *	278.1 ± 135.7	311 ± 109.7	0.920
**B. AFTER EDUCATION**			
Vitamins	SG avg ± SD (n = 31)	CG avg ± SD (n = 18)	*p*
Vitamin A (µg)	1034.5 ± 495.8	1308 ± 530.6	0.391
Retinol (µg)	344.3 ± 133.5	375 ± 120.4	0.344
Beta carotene (µg)	4152.8 ± 2987	5638.2 ± 3090	0.090
Vitamin E (mg) †	8.1 ± 3	10.1 ± 2.5	**0.038**
Thiamine (mg)	1.2 ± 0.3	1.4 ± 0.5	0.495
Riboflavin (mg)	1.6 ± 0.4	1.8 ± 0.5	0.708
Niacin (mg)	19.9 ± 5.7	22.8 ± 9.7	0.333
Vitamin B6 (mg)	2.1 ± 0.4	2.3 ± 0.8	0.333
Vitamin C (mg)	100.4 ± 64.7	133.6 ± 64.7	0.166
Vitamin B12 (µg)	3.3 ± 1	4.2 ± 2.3	0.500
Vitamin D (µg) *	3.5 ± 1.5	4.6 ± 3.4	0.878
Folates (µg )*	295.9 ± 100.2	322.2 ± 84.2	0.118

SG—study group; CG—control group; bold—statistically significant differences. Folates and vitamin D were predefined as primary endpoints (*). All other vitamins were considered secondary endpoints and analyzed exploratorily with false discovery rate (FDR) correction using the Benjamini–Hochberg procedure; statistically significant results after FDR correction are marked with a dagger (†).

**Table 6:** Mineral consumption comparison.

**A. BEFORE EDUCATION**			
Minerals	SG avg ± SD (n = 44)	CG avg ± SD (n = 18)	*p*
Sodium (mg)	4328.8 ± 1301.5	4523 ± 1914.6	0.716
Potassium (mg)	3270.5 ± 1317	3828.4 ± 1634.5	0.190
Calcium (mg)	947.3 ± 492.7	958.4 ± 542.6	0.332
Phosphorus (mg)	1423.7 ± 565.8	1497.7 ± 545.1	0.550
Magnesium (mg)	351.3 ± 114.8	387.5 ± 122.5	0.235
Iron (mg) *	12 ± 8.3	12.6 ± 5.2	0.340
Zinc (mg)	10.6 ± 4.4	10.6 ± 3.9	0.834
Copper (mg)	1.2 ± 0.5	1.3 ± 0.4	0.288
Manganese (mg)	5 ± 2.7	4.9 ± 2.5	0.834
Iodine (µg) *	107.8 ± 51.7	105.1 ± 95	0.625
**B. AFTER EDUCATION**			
Minerals	SG avg ± SD (n = 31)	CG avg ± SD (n = 18)	*p*
Sodium (mg)	2910.5 ± 947.6	3480.6 ± 956.1	0.982
Potassium (mg)	3465.8 ± 637.6	3656.3 ± 1426.7	0.878
Calcium (mg)	985.3 ± 248.5	1079.6 ± 243.1	0.912
Phosphorus (mg)	1378.2 ± 221.6	1440.2 ± 396.6	0.741
Magnesium (mg)	343.1 ± 75.4	367.5 ± 109.6	0.741
Iron (mg) *	12.4 ± 2.4	12.5 ± 3.9	0.524
Zinc (mg)	10.3 ± 2.6	10.9 ± 3.1	0.416
Copper (mg)	1.2 ± 0.4	1.3 ± 0.3	0.441
Manganese (mg)	4.6 ± 1.8	4.5 ± 1.8	0.281
Iodine (µg) *	121.1 ± 54.6	118.9 ± 50.9	0.202

SG—study group; CG—control group; bold—statistically significant differences; *—*p* < 0.05, primary endpoint; no secondary endpoints reached significance after FDR correction.

## Data Availability

The original contributions presented in this study are included in the article. Further inquiries can be directed to the corresponding author..

## Abbreviations

The following abbreviations are used in this manuscript:

AI	adequate intake
BMI	body mass index
DHA	docosahexaenoic acid
EAR	average group requirement
EPA	eicosapentaenoic acid
MUFA	monounsaturated fatty acid
PUFA	polyunsaturated fatty acid
RDA	recommended dietary allowances
SFA	saturated fatty acids
